# Establishment of a new genus, *Brephallus* Wang et al., gen. nov. (Blattodea, Blaberidae, Epilamprinae) based on two species from *Pseudophoraspis*, with details of polymorphism in species of *Pseudophoraspis*

**DOI:** 10.3897/zookeys.785.26565

**Published:** 2018-09-19

**Authors:** Zhenzhen Wang, Qiongyao Zhao, Weijun Li, Yanli Che, Zongqing Wang

**Affiliations:** 1 Institute of Entomology, College of Plant Protection, Southwest University, Beibei, Chongqing 400715, China Southwest University Chongqing China

**Keywords:** China, sexual dimorphism, species delimitation, taxonomy, cockroaches

## Abstract

*Brephallus* Wang et al., **gen. n.** is established as a genus distinct from *Pseudophoraspis* Kirby, 1903 because of the lack of a well-developed apical outgrowth on sclerite L2D and substantial genetic differences. Two species are transferred to the new genus from *Pseudophoraspis*, i.e., *Brephallusfruhstorferi* (Shelford, 1910), **comb. n.** and *Brephallustramlapensis* (Anisyutkin, 1999), **comb. n.** We provide a detailed generic diagnosis of *Brephallus* Wang et al., **gen. n.** Based on COI data, males, females and nymphs of three *Pseudophoraspis* species (*P.clavellata* Wang et al., 2013, *P.recurvata* Wang et al., 2013 and *P.kabakovi* Anisyutkin, 1999) were successfully matched. The former two are sexually dimorphic with macropterous males and micropterous females. Photos of the species from China are presented.

## Introduction

*Pseudophoraspis* Kirby, 1903 is a genus of Epilamprinae cockroaches from South-east Asia whose taxonomy and biogeography were recently discussed by Wang et al. (2013). They exhibit some parental care behaviors rare among cockroaches (Tallamy and Wood 1986; Clutton-Brock 1991). *Pseudophoraspisnebulosa*, the type species of the genus, has offspring that cling ventrally to the parent’s body after hatching and feed on their mother’s bodily secretions (Nalepa and Bell 1997). Yet, owing to the lack of research on *Pseudophoraspis*, this behavior in other members of the genus remains unknown.

Currently, the genus is composed of 18 species (Beccaloni 2014). According to original descriptions, 15 species are from South-east Asia (Cambodia, Thailand, Vietnam and Malaysia), and three from South China (Yunnan, Hainan). Among these, internal male genitalia are known for only 13 species (Anisyutkin 1999; Anisyutkin 2005; Wang et al. 2013). Meanwhile, only the external morphology of the remaining five species has been described, four of which are based on female specimens (Walker 1868; Hanitsch 1925, 1933).

In the past, some external morphological characters have been used to diagnose *Pseudophoraspis* (e.g. male and female with fully-developed tegmina and wings, and head entirely covered by the pronotum; Kirby 1903; Shelford 1910; Hanitsch 1915; Princis 1958; Wang et al. 2013). Additionally, the genus has been identified by the apical part of sclerite L2D having a well-developed apical outgrowth (Anisyutkin 1999; Wang et al. 2013). Yet, two species, *P.fruhstorferi*Shelford 1910 and *P.tramlapensis*Anisyutkin 1999 are distinctively different from their congeners by the absence of this genital character (Anisyutkin 1999; Wang et al. 2013). Anisyutkin (1999) mentioned that *P.fruhstorferi* and *P.tramlapensis* were included conditionally in *Pseudophoraspis*. Wang et al. (2013) subdivided this genus into two species groups, the *fruhstorferi* group and the *gorochovi* group, but only according to pronotal characteristics and without information on the females of the *gorochovi* group. Males, females and nymphs in this genus from the same locality are difficult to match accurately (Wang ZZ, pers. obs.). Sexual dimorphism can exaggerate male-female differences to the extent that the sexes appear to be entirely different species. In the genera *Escala* Shelford, 1906 and *Robshelfordia* Princis, 1954, for example, most females have micropterous tegmina that are reduced to small lateral pads without wings, and in the genus *Laxta* Walker, 1868, the females are apterous. But the males of these three genera usually have fully-developed tegmina and wings (Roth 2003).

The commonly-adopted, standard COI sequence has proven to be highly informative and successful in resolving problems of polymorphism, sexual dimorphism and identification of nymphs in cockroaches (Evangelista et al. 2013; Yue et al. 2014; Che et al. 2017; Bai et al. 2018). These issues highlight the need for determining the taxonomic status of *P.fruhstorferi* and *P.tramlapensis*, and clarifying approaches toward solving sexual dimorphism in cockroach species.

In this study, *Brephallus* Wang et al., gen. n. is established for two species, *Brephallusfruhstorferi* (Shelford, 1910), comb. n. and *Brephallustramlapensis* (Anisyutkin, 1999), comb. n. A combination of newly generated and publicly available molecular data (COI) has been used to aid in associating adult sexual morphs and juveniles. Additionally, this study adds to the knowledge of cockroach diversity in China.

## Material and methods

### Specimen collection and morphological study

In this study, 32 specimens were collected at night with the help of headlight from dead leaves of grasses or shrubs in the litter layer. Other specimens were mostly collected with a net in daytime. Voucher specimens are deposited in the Institute of Entomology, College of Plant Protection, Southwest University (SWU), Chongqing, China.

Terminologies used for male genitalia mainly follow Klass (1997) and Anisyutkin (2014). The apical part of an abdomen was removed and macerated in 10% NaOH and observed in glycerin jelly using a Motic K400 stereomicroscope. The dissected genitalia were preserved in glycerin jelly. Specimens were photographed using a Canon50D with a Canon EF 100mm f/2.8L Macro IS USM Macro USM lens, and stacked with Helicon Focus software. All photos and images were edited with Adobe Photoshop CS5. Male adults were identified to species mainly based on morphological characters, including the apical part of sclerite L2D, the macula on the head, depressions and punctuation on the pronotal disk, and wing size.

### Phylogenetic data collection and analysis

Tissue samples from adult females and nymphs were used directly for PCR analysis and DNA sequencing. The hind legs were used for DNA extraction. Other body parts were stored in 95% ethanol as voucher specimens. In total, 32 specimens were used for COI sequencing in this study and all sequences are deposited at the National Center for Biotechnology Information GenBank (Table 1).

**Table 1.** Specimens for which COI DNA barcodes were sequenced.

Species	Specimen voucher	Sequence ID	Location (China)	Accession Number
*** P. clavellata ***	I01.1M	RhicClav01	Xishuangbanna, Yunnan	MH755944
	I01.2M	RhicClav03	Pu’er, Yunnan	MH755945
	I01.2F	RhicClav04	Pu’er, Yunnan	MH755946
	I01.3M	RhicClav02	Xishuangbanna, Yunnan	MH755947
	I01.4M	RhicClav05	Pu’er, Yunnan	MH755948
	I01.5N	RhicClav06	Xishuangbanna, Yunnan	MH755949
*** P. recurvata ***	I02.1M	RhicRecu01	Changjiang, Hainan	MH755950
	I02.2F	RhicRecu05	Sanya, Hainan	MH755951
	I02.3M	RhicRecu03	Baoting, Hainan	MH755952
	I02.3F	RhicRecu04	Baoting, Hainan	MH755953
	I02.4M	RhicRecu02	Changjiang, Hainan	MH755954
	I02.5F	RhicRecu06	Sanya, Hainan	MH755955
*** P. kabakovi ***	E04.1F	RhicKaba02	Menglun, Yunnan	MH755937
	E04.1M	RhicKaba01	Menglun, Yunnan	MH755938
	E04.2F	RhicKaba04	Xishuangbanna, Yunnan	MH755939
	E04.2N	RhicKaba05	Menglun, Yunnan	MH755940
	E04.2M	RhicKaba03	Xishuangbanna, Yunnan	MH755941
	E04.3M	RhicKaba06	Menglun, Yunnan	MH755942
	E04.4F	RhicKaba07	Menglun, Yunnan	MH755943
*** B. fruhstorferi ***	E01.1M	PseuFruh01	Jianfengling, Hainan	MH755924
	E01.2M	PseuFruh02	Limushan, Hainan	MH755925
	E01.4N	PseuFruh04	Diaoluoshan, Hainan	MH755926
	E01.5F	PseuFruh05	Jianfengling, Hainan	MH755927
	E01.5M	PseuFruh06	Jianfengling, Hainan	MH755928
	E01.7F	PseuFruh09	Wuzhishan, Hainan	MH755929
	E01.8F	PseuFruh11	Yinggeling, Hainan	MH755930
	E01.8M	PseuFruh10	Yinggeling, Hainan	MH755931
	E01.9F	PseuFruh12	Yinggeling, Hainan	MH755932
	E01.9M	PseuFruh13	Yinggeling, Hainan	MH755933
*** B. tramlapensis ***	E02.1M	PseuTram01	Damingshan, Guangxi	MH755934
	E02.2M	PseuTram02	Dayaoshan, Guangxi	MH755935
	E02.3M	PseuTram03	Mangshan, Hunan	MH755936

DNA extraction, PCR amplification and sequencing follow Bai et al. (2018). COI specific primers were used: LCO1490 (GGTCAACAAATCATAAAGATATTGG); and HCO2198 (TAAACTTCAGGGTGACCAAAAAATCA).

PCR products were sent to BGI Technology Solutions Company Limited (BGI-Tech) (Beijing, China) for sequencing using the aforementioned primers.

A total of 48 COI sequences were analyzed (32 new sequences from this study, and 14 cockroach sequences and 2 mantis sequences downloaded from GenBank; Table 2). All COI sequences were aligned using MUSCLE 3.8 (Edgar 2004) and adjusted visually after translation into amino acid sequences. Intraspecific and interspecific genetic divergence values were quantified based on the Kimura 2-parameter (K2P) distance model (Kimura 1980), and variance was estimated by using bootstrap method with 1000 bootstrap replications in MEGA 6.0.6 (Tamura et al. 2013). Phylogenetic analysis was done using Maximum Likelihood (ML) in RAxML (Stamatakis et al. 2008) following the GTR GAMMA model with 1000 bootstrap replicates.

**Table 2.** Ectobiidae and Mantodea (outgroup) used in this study.

Species	Family	Accession number	Reference
*** Sorineuchora bivitta ***	Ectobiidae	KY349592, KY349593	Che et al. 2017
*** Sorineuchora nigra ***	Ectobiidae	KY349516-KY349522	Che et al. 2017
*** Allacta ornata ***	Ectobiidae	KY349665	Che et al. 2017
*** Balta jinlinorum ***	Ectobiidae	KY349666-KY349669	Che et al. 2017
*** Mantis religiosa ***	Mantidae	KR148854, KM529415	Hebert et al. 2016, Dewaard et al. (Unpublished)

## Results

### Phylogenetic analysis based on COI data

In this study, we acquired 32 COI sequences whose length, excluding primers, was 658bp each. All of the new sequences have been deposited in GenBank with accession numbers MH755924 to MH755955 (Table 1). The COI region we sequenced had a relatively high AT content (65.8%), with an average nucleotide composition of A = 30.3%, T = 35.5%, C = 18.3%, and G = 15.9%. Sequence analysis revealed that 156 (23.71%) sites were variable, of which 148 (22.49%) sites were parsimoniously informative.

**Figure 1.**
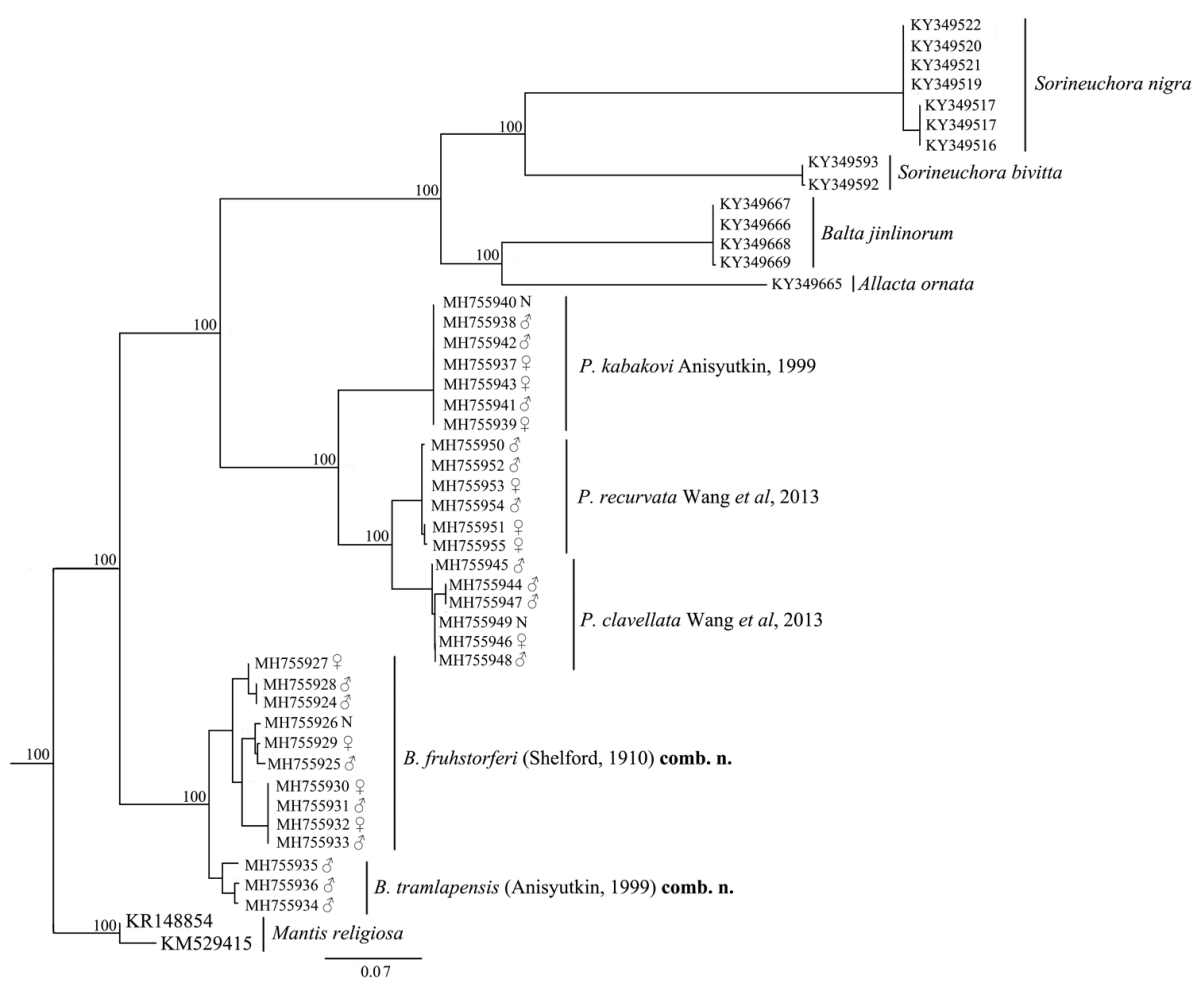
Maximum likelihood (ML) tree derived from COI gene analysis following GTR GAMMA model with 1000 bootstrap replicates. The bootstrap support are all 100%, in this phylogenetic tree.

The ML phylogeny of the COI data revealed that clades from the same species, including females and nymphs, constitute monophyletic groups with very strong bootstrap values (all MLB = 100) (Figure 1). Three recognized major lineages of *Pseudophoraspis* (*P.clavellata*, *P.recurvata* and *P.kabakovi*) are recognized, and cluster with the ectobiids *Sorineuchora*, *Allacta* and *Balta*, with high support values, but are more distant from the other two Epilamprinae, *P.fruhstorferi* and *P.tramlapensis*.

### Establishment of *Brephallus* Wang et al., gen. n.

*Pseudophoraspisfruhstorferi* and *P.tramlapensis* are easily distinguished from other congeners by the apical part of sclerite L2D lacking a well-developed apical outgrowth (Anisyutkin 1999; Wang et al. 2013). Other diagnostic morphological characters of these two species compared with other *Pseudophoraspis* members are shown in Table 3. Therefore, these two species are moved to *Brephallus* Wang et al., gen. n. (i.e., *Brephallusfruhstorferi* (Shelford, 1910), comb. n., and *Brephallustramlapensis* (Anisyutkin, 1999), comb. n.). In addition, these two species were recovered as sister groups and show a close relationship to each other, but were distant from the other three *Pseudophoraspis* species (Figure 1).

#### Genus Brephallus gen. n.

Taxon classificationAnimaliaBlattodeaBlaberidae

http://zoobank.org/6023C59C-4D25-4730-9FB1-90FEAA7CD51F

**Figure 2.**
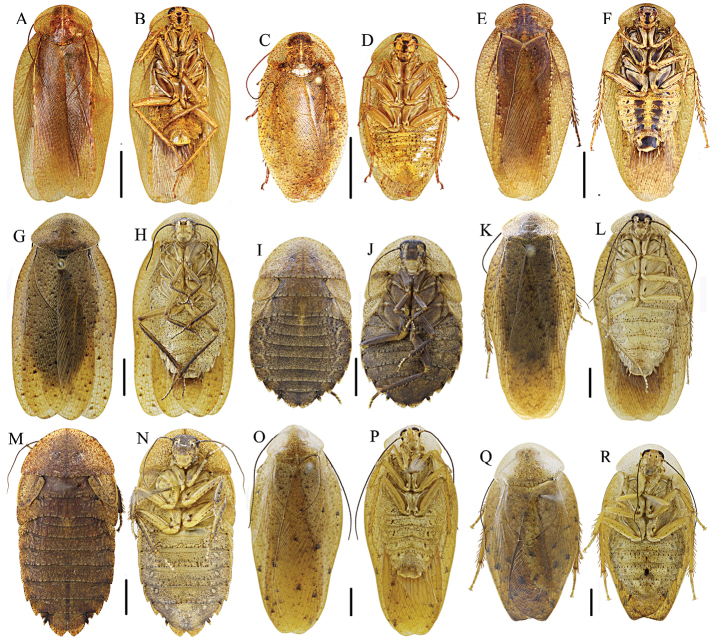
**A–D***Brephallusfruhstorferi* (Shelford, 1910) **comb. nov.** (male **A–B** female **C–D**) **E–F***Brephallustramlapensis* (Anisyutkin, 1999) **comb. nov.** (male) **G–J***P.recurvata* (male **G–H** female **I–J**) **K–N***P.clavellata* (male **K–L** female **M–N**) **O–R***P.kabakovi* (male **O–P** female **Q–R**). Scale bars = 10 mm (**A–F**) Scale bars = 5 mm (**G–R**).

**Figure 3.**
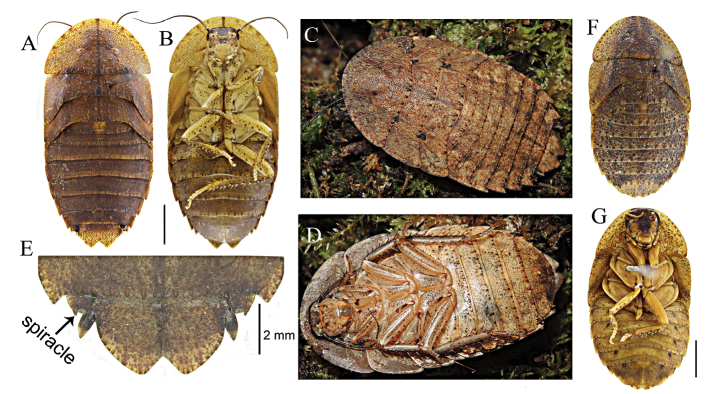
**A–B***P.kabakovi* (nymph) scale bars = 5 mm **C–D***P.clavellata* (nymph) **E** abdomen of female of *P.clavellata***F–G***Brephallusfruhstorferi* (Shelford, 1910) **comb. nov.** (nymph).

**Figure 4.**
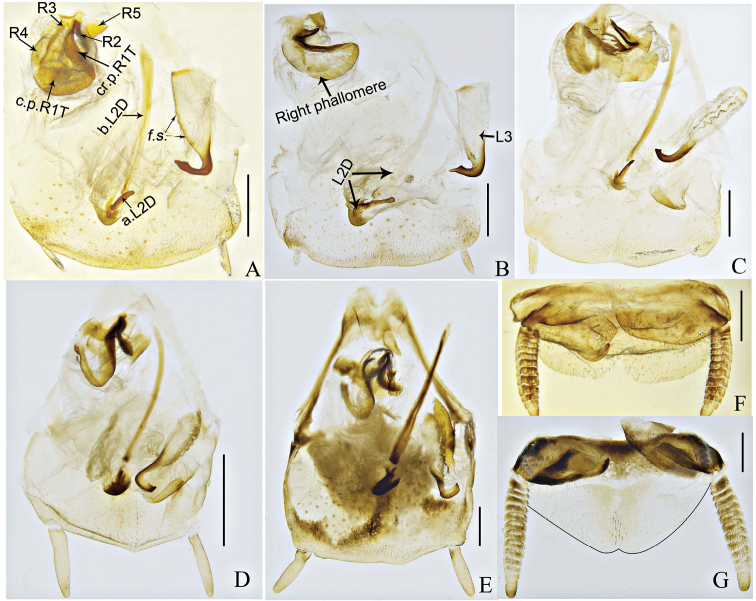
Male genitalia of *Pseudophoraspis* and *Brephallus* Wang et al., **gen. nov. A***P.recurvata***B***P.clavellata***C***P.kabakovi***D***Brephallusfruhstorferi* (Shelford, 1910) **comb. nov. E***Brephallustramlapensis* (Anisyutkin, 1999) **comb. nov. F** supra-anal plate of *P.recurvata***G** supra-anal plate of *Brephallustramlapensis* (Anisyutkin, 1999) **comb. nov.** (Scale bars = 1 mm).

**Figure 5.**
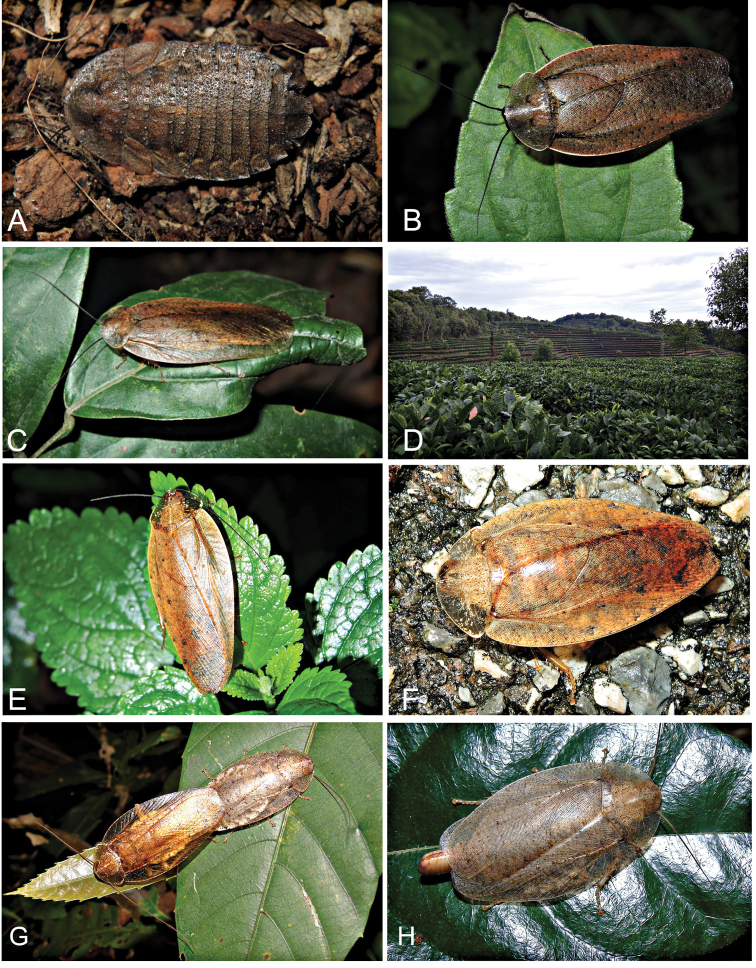
**A** female of *P.recurvata* from Hainan Province. This specimen, collected as a nymph in 8 April 2015, was reared at Southwest University by Lu Qiu and adult emergence occurred in 21 May 2015 **B** male of *P.recurvata* from Hainan Province **C** male of *P.clavellata* from Yunnan Province **D** habitat of *P.clavellata***E** male of *P.kabakovi* from Yunnan Province **F** female of *P.kabakovi* from Yunnan Province. **A–F** Photographed by Lu Qiu **G–H***Brephallusfruhstorferi* (Shelford, 1910) **comb. nov.** from Hainan Province (Photographed by Xin-Ran Li).

Figures 2A–F , 3F–G , 4D–E , 4G , 5G–H

##### Species included.

*Brephallusfruhstorferi* (Shelford, 1910), comb. n., *Brephallustramlapensis* (Anisyutkin, 1999), comb. n.

##### Type species.

*Pseudophoraspisfruhstorferi* Shelford, 1910, by present designation.

##### Generic diagnosis.

Coloration brownish yellow. Pronotum smooth, completely covering vertex, anterior margin curved and posterior margin obtusely produced. Tegmina and wings fully developed in both sexes, entirely covering abdomen, tegmina about twice as long as broad, apices rounded (Figure 2A–F). Hind metatarsus shorter than succeeding tarsal segments combined, with two equal rows of spines along most of its length, 2^nd^–4^th^ segments with large euplantulae. Supra-anal plate and hypandrium nearly symmetrical, posterior margin emarginate near mid-line (Figure 4G).

Male genitalia (Figure 4D–E). Right phallomere similar to that in *Morphna*, *Opisthoplatia*, and *Rhabdoblatta* with well-developed caudal part of sclerite R1T subrectangular in shape, R2 rounded, R3 well developed, widened caudally and fused with R5. Sclerite L2D similar to *Rhabdoblatta*, divided into basal and apical parts, basal part rod-like, apical part more or less rounded, cap-shaped, but with more bristles. Sclerite L3 with terminal rectangular apex pointed and folded, scattered with bristles.

The new genus differs from other genera of Epilamprinae as follows: 1) male tegmina about twice as long as broad (Figure 2A, E); 2) facial part of head with large brown spot from ocellus to clypeus, basal margin of ocellus with brown spot (Figure 2B, D, F); 3) one third of radius vein of tegmen from base yellowish white (Figure 2A, C, E); 4) sclerite L3 with terminal rectangular, apex pointed (Figure 4D–E).

##### Etymology:

We propose the name *Brephallus*, based on the composition of two Latin words (“*brevis*” and “*phallus*”) meaning “short phallomere”, in reference to the short L2D sclerite of the male genitalia.

##### Remarks.

This genus differs from *Pseudophoraspis* in the apical part of sclerite L2D without a well-developed apical outgrowth. Meanwhile, the mean sequence divergence among species of *Brephallus* and *Pseudophoraspis* ranged from 15.2% to 18.8%, larger than that of congeners (Table 4). Although *Brephallusfruhstorferi* and *B.tramlapensis* only have the mean interspecific genetic distance of 4.1% (Table 4) between them, they show distinct morphological differences as follows: 1) mid-abdomen of *B.tramlapensis* (Anisyutkin, 1999) has two brown stripes while *B.fruhstorferi* (Shelford, 1910) lacks stripes (Figure 2A–F); and 2) the apical part of sclerite L2D of *B.tramlapensis* (Anisyutkin, 1999) is large and long, with a protrusion in the middle (Figure 4E) while in *B.fruhstorferi* (Shelford, 1910) it is short, without a protrusion in the middle (Figure 4D).

#### Pseudophoraspis

Taxon classificationAnimaliaBlattodeaBlaberidae

Kirby, 1903

Figures 2G–R , 3A–E , 4A–C , 4F , 5A–F

##### Type species:

*Epilampranebulosa* Burmeister, 1838.

The species *P.clavellata* and *P.recurvata* exhibit sexual dimorphism (male with developed tegmina and wings, females with tegmina reduced to lateral scales and wings absent) (Figure 2G–N); therefore, we provide below supplementary information on the nymphs and females.

##### Generic description.

Body slender, general color yellowish brown, head entirely covered by pronotum. Pronotum with numerous brown spots, smooth, without or with scattered punctuation. Male with fully-developed tegmina and wings, female with tegmina reduced to lateral scales without wings or with fully-developed tegmina and wings (Figure 2G–R). Hind metatarsus shorter than other tarsal segments combined, with small apical euplantulae along its lower margin, with spinules, euplantulae occupying less than half of its length, with two equal rows of spines along most of its length. Tarsal claws symmetrical and unspecialized. Supra-anal plate semicircular, meso-posterior margin emarginate (Figure 4F).

Male genitalia (Figure 4A–C). Right phallomere with well-developed caudal sclerite, R1T subrectangular in shape (Figure 4A–C “c.p.R1T”), R2 rounded, R3 weakly sclerotized, without branch, narrowed caudally. Sclerite L2D divided into basal and apical parts, basal part rod-like, apical part with well-developed apical outgrowth (Figure 4A–C “a.L2D”), with bristles. Sclerite L3 with apex pointed and folded structure scattered with bristles (Figure 4A–C “*f.s.*”).

##### Remarks.

Wang et al. (2013) subdivided the Chinese *Pseudophoraspis* into two species groups: the *fruhstorferi* group and the *gorochovi* group, but the latter lacked information on females. The *fruhstorferi* group currently includes three species: *P.fruhstorferi* Shelford, 1910, *P.tramlapensis* Anisyutkin, 1999 and *P.kabakovi* Anisyutkin, 1999. Because we have transferred the former two species to the new genus, *Brephallus* Wang et al., gen. n., the *fruhstorferi* group is renamed as *nebulosa* group. Some diagnostic characters between the *nebulosa* group and the *gorochovi* group are shown in Table 3.

**Table 3.** Diagnostic morphological characters among *Brephallus* Wang et al., gen. n., the *nebulosa* group and the *gorochovi* group of *Pseudophoraspis*.

Species	Characters						
	I	II	III	IV	V	VI	VII
*Brephallus* Wang et al., gen. n.	0	1	1	2	0	1	0
*Pseudophoraspisnebulosa* group	0	1	0	0	1	0	1
*Pseudophoraspisgorochovi* group	1	0	0	1	1	0	1

**I** depressions and punctuation on pronotum present (1), absent (0) **II** females with tegmina and wings fully-developed (1), tegmina reduced to lateral scales without wings (0) **III** male tegmina about twice as long as broad (1), tegmina length more than twice as broad (0) **IV** facial part of head with large brown spot from ocellus to clypeus, basal margin of ocellus with brown spot (2), vertex to basal margin of ocellus with brown spot (1), inside and basal margin of ocellus with brown round spot (0) **V** female supra-anal plate with posterior margin distinctly exceeding posterior margin of subgenital plate (1), not beyond (0)**VI** R3 well-developed, widened caudally and fused with R5 of right phallomere (1), R3 not widened caudally and fused with R5 (0) **VII** the apical part of sclerite L2D with well-developed apical outgrowth (1), without (0)

The mean interspecific sequence divergence among the three *Pseudophoraspis* members ranged from 4.1% to 9.0% (Table 4), but there are distinguishing differences among them, as described below.

**Table 4.** The variance of the underlying distribution of distances calculated by using K 2–P model and bootstrap method respectively in MEGA.

Species	* B. fruhstorferi *	* B. tramlapensis *	* P. kabakovi *	* P. clavellata *	* P. recurvata *
* Brephallus fruhstorferi *	–	–	–	–	–
* Brephallus tramlapensis *	0.041±0.007	–	–	–	–
* P. kabakovi *	0.188±0.018	0.172±0.017	–	–	–
* P. clavellata *	0.174±0.017	0.152±0.016	0.090±0.012	–	–
* P. recurvata *	0.171±0.017	0.157±0.016	0.087±0.011	0.041±0.008	–

### *Pseudophoraspisgorochovi* group

**Species included here.***P.clavellata* Wang et al., 2013; *P.recurvata* Wang et al., 2013; *P.incurvata* Wang et al., 2013; and *P.gorochovi* Anisyutkin, 1999.

#### Pseudophoraspis recurvata

Taxon classificationAnimaliaBlattodeaBlaberidae

Wang et al., 2013

Figures 2G–J , 4A , 4F , 5A–B

##### Note.

Wang et al. (2013) described the male of *P.recurvata* including detailed information on male genital structures (Figures 2G–H, 4A, 4F, 5B). The description of the female is provided here.

##### Material examined.

China: Hainan: five males and one female, Baoting County, 2013.V.2, coll. Yan Shi and Shun-Hua Gui; three males, Changjiang County, Qicha Township, 2015.IV.28, coll. Lu Qiu and Qi-Kun Bai; one male and two females, Sanya City, Liupan Village, 2015.IV.8, coll. Lu Qiu and Qi-Kun Bai; two males (holotype and paratype), Baoting County, 1959.VII.10, coll. Yi-Chuan Hu. China: Guangxi: one male (paratype), Mt. Daqingshan, 1958.IX, coll. Yi-Xin Xu.

##### Female description.

(Figures 2I–J, 5A). Body brownish-yellow. Vertex, eyes and between the antennal sockets black-brown. Ocellar spots pale yellowish. Antennae, legs, thorax and abdomen brown. Maxillary palp with 1^st^–2^nd^ segments pale yellowish and 3^rd^–5^th^ segments brown. Cerci brown with apical segment yellow.

Head longer than wide. Interocular space slightly less wide than interocellar space, ocellar spots rather small, eyes elongate. Antennae short, not reaching to half length of body, first segment of flagellum twice length of next segment; interantennal portion of frons concave. Frons moderately punctuated; clypeus and labrum unmarked. Pronotum covering vertex of head, anterior margin arcuate, posterior margin truncate, with scattered punctuation and a pair of impressions on disc. Thoracic and abdominal tergites with small tubercles and longitudinal inflations along posterior margins. Tegmina reduced to lateral scales, with nearly indistinct venation, veins reduced, wings absent. Anterior margin of fore femur type B, with six large spines and one single apical spine. Tibial spines well developed. 3^rd^–7^th^ abdominal tergites with paired rounded impressions. Hind metatarsus with spines along most of its length, equal to remaining joints, tarsal spines absent. Tarsal claws symmetrical, simple, arolia very small. Supra-anal plate transverse, beyond the subgenital plate, hind margin with a medial V-shaped excavation. Hypandrium widely rounded, caudal margin arcuate. Cerci abbreviated, apex blunt.

##### Variation.

Morphology of paratypes is same as female type described above, but with following variation: five to six large spines scattered along anterior margin of fore femora; color of clypeus, labrum and abdomen tergites brown or yellow. Overall length: 20.1 ± 0.2 mm; head length × width: 3.6 ± 0.1 mm × 2.9 ± 0.1 mm; pronotum length × width: 6.2 ± 0.1 mm × 10.7 ± 0.1 mm.

##### Known geographic range.

China (Hainan, Guangxi).

#### Pseudophoraspis clavellata

Taxon classificationAnimaliaBlattodeaBlaberidae

Wang et al., 2013

Figures 2K–N , 3C–E , 4B , 5C–D

##### Note.

Wang et al. (2013) described the male of *P.clavellata* including the male genitalic structures (Figures 2K–L, 4B and 5C). Description of the female and nymph is provided here.

##### Material examined.

China: Yunnan: Thirty males and one female, Pu’er City, Meizi Lake, 2016.V.20, coll. Lu Qiu and Zhi-Wei Qiu; two males, Jinhong City, Dadugang, 2014.VI.29, coll. Conlin McCat (= Xin-Ran Li) and Hong-Guang Liu; one nymph, Xishuangbanna, Menghai County, Bulong Natural Reserve, 2017.I.31, coll. Jian-Yue Qiu and Hao Xu; male (holotype), Xishuangbanna, 1981.V.27-30, coll. Zhi-Gang Zheng.

##### Female description

(Figures 2M–N, 3E). Identical to the female of *P.recurvata* but body larger; in addition, legs, venter of thorax and abdomen yellow.

##### Female measurements.

Overall length 28.1 mm; head length × width: 3.8 mm × 3.7 mm; pronotum length × width: 7.0 mm × 12.5 mm.

##### Nymph

(Figure 3C–D). Body flattened. Identical to adult female but lacking wings.

##### Known geographic range.

China (Yunnan).

### *Pseudophoraspisnebulosa* group

According to the original descriptions of male genitalia of these species: *P.kabakovi* Anisyutkin, 1999, *P.marginata* Anisyutkin, 1999, *P.grigorenkoi* Anisyutkin, 1999, *P.argillacea* Anisyutkin, 1999, *P.truncatulus* Anisyutkin, 1999, *P.buonluoiensis* Anisyutkin, 1999 and *P.doroshenkoi* Anisyutkin, 2005, the apical part of sclerite L2D has a well-developed apical outgrowth, pronotum smooth without punctuation, and both male and female have fully developed tegmina and wings. We therefore assign these seven species to the *Pseudophoraspisnebulosa* group.

#### Pseudophoraspis kabakovi

Taxon classificationAnimaliaBlattodeaBlaberidae

Anisyutkin, 1999

Figures 2O–R , 3A–B , 4C , 5E–F

##### Note.

The male of *P.kabakovi* was described (Figures 2O–P, 4C, 5E) by Anisyutkin (1999) and Wang et al. (2013), but little was known about the female and nymph until now.

##### Materials examined.

China: Yunnan: One male, Xishuangbanna, 1974.IV.13, coll. Yao Zhou and Feng Yuan; twenty males, five females and one nymph, Xishuangbanna, Menglun Town, 2016.V.27, coll. Lu Qiu and Zhi-Wei Qiu; one male and two females, Xishuangbanna, Mengla County, Wangtianshu, 2016.V.23, coll. Lu Qiu and Zhi-Wei Qiu.

##### Female description

(Figures 2Q–R, 5F). Body yellowish brown. Eyes and antennae black, ocellar spots pale yellow. Pronotum with dense small brown spots. Tegmina with scattered large black spots. Abdominal sterna with small and fewer large black dots, large black dots along the hind margins of the segments. Cerci brown.

Similar to male in general appearance, but shorter and convex. Tegmina and wings shorter than in males. Fore femur with six spines along anterior margin and one single apical spine. Hind metatarsus with two rows of spines along most of its length. Claws symmetrical, simple; arolium well developed. Abdominal terga unspecialized. Supra-anal plate caudal margin with a medial V-shaped excavation. Hypandrium posterior margin emarginate near mid-line.

##### Female measurements.

Overall length 32 ± 0.2 mm; head length × width: 4.2 ± 0.1 mm × 3.6 ± 0.1 mm; pronotum length × width: 8.3 ± 0.2 mm × 12.1 ± 0.2 mm; tegmina length × width: 25.4 ± 0.1 mm × 10.3 ± 0.2 mm.

##### Nymph.

Identical to adult females of *P.recurvata* and *P.clavellata* except for undeveloped wing (Figure 3A–B).

##### Known geographic range.

China (Yunnan); Vietnam.

## Discussion

Five Epilamprine species were identified mainly on the basis of morphological and male genitalia data. Due to the apical part of sclerite L2D lacking a well-developed apical outgrowth, two species of *Pseudophoraspis* are transferred to *Brephallus* Wang et al., gen. n.

Our molecular results show two members of the *Pseudophoraspisgorochovi* group, *P.recurvata* Wang et al., 2013 and *P.clavellata* Wang et al., 2013, collected in China were sexually dimorphic. However, the other species group within this genus, *P.nebulosa* group, is not sexually dimorphic. As we have applied it, and as others have shown (Che et al. 2017; Bai et al. 2018; Evangelista et al. 2013), the integration of morphological and DNA-based approaches is useful for cockroach species identification and to supplement morphological keys, which are typically limited to adult male morphological characters.

## Supplementary Material

XML Treatment for Brephallus

XML Treatment for Pseudophoraspis

XML Treatment for Pseudophoraspis recurvata

XML Treatment for Pseudophoraspis clavellata

XML Treatment for Pseudophoraspis kabakovi
